# Embryotomy—Novel Expedient

**Published:** 1874-12

**Authors:** 


### Editorial.

A CORRESPONDENT WRITES US: "I was called last March to see a negro woman who needed obstetrical aid. Without entering into the particulars of the case, I desire merely to speak of the means used to effect delivery. I was three miles from home, and having no obstetrical instruments with me, I succeeded satisfactorily in opening the fœtal head with my pocket knife, the blade and handle of which measured seven and one-quarter inches in length. Having done this, and enlarged the opening with an ordinary pair of tooth forceps, I inserted the index finger of my right hand into the orifice, and, without exerting much force, succeeded in effecting delivery."

For readiness in emergencies, and cunning ingenuity in seeking out handy means of accomplishing needful ends, commend us to the rural practitioners of our gloomy, malarial jungles and airy, mountain fastnesses. We doubt not the necessity for the embryotomy cited by our correspondent was both manifest and imperative; and while we make it the occasion for a few remarks upon the subject of embryotomy in general, we desire to be understood that we do not call this in question.

Time was when it was universally esteemed a masterly stroke of professional ability, in critical cases, to make *vital issues* with the sick. "In your extremity I give you this potential dose; six hours, at most, must determine your fate; upon the silver thread of this masterly remedy hangs your life or your death; if this delicate pendulum shall, by happy chance, vibrate towards you, you shall surely live; if, unhappily, it vibrates from you, your doom is thereby inevitably sealed. Gape, sinner, and swallow thy doom!" Such was once the dictum of the savans of the powdered wig, the golden snuff-box, and the gold-headed cane. Fortunately, as we would fain believe, for human longevity, this golden age of professional pomposity and power is now forever embalmed in the gums and spices of medical history, never more to resume its sway upon the earth. In our day, the controlling power of what we are accustomed so dimly to define by the terms vital force, vis medicatrix, etc., is becoming more and more distinctly recognized as a firm and solid foundation upon which to erect our slender, and more or less temporary, therapeutical superstructures. Whatever may be the fate of our human thought and labor, and their outcome, in the grand mutations of coming ages, we may rest assured that the irrevocable vital laws, which have been impressed upon our being from its very incipiency, will continue their undisputed sway until the sounding of the last trump shall herald the extinction of our race from the earth.

In obstetrical, as well as in our medical ministrations, it is wise to bear in mind that our professional office calls upon us to assist the efforts of Nature in the conservation of life; this is our chief duty. Whilst it cannot be denied that there are circumstançes under which we are compelled to elect between the life of the mother and that of her offspring, we should not forget that it is by the stress of *necessity* only that we can find our justification in these extreme cases. We must not forget, too, that the statistics of embryotomy which are accessible to us in our standard works are made up, for the most part, from the records of hospitals and lying-in-asylums, located in the great commercial centres of densely populated districts, where the sanitary surroundings, as well as the physical development of the *accouche*, herself, are generally defective, and markedly in contrast with the conditions which favor us in our private practice; and hence the necessity to interpose the hand of destruction must occur with much greater frequency than in our own cases. As for ourselves, we are disposed to view with much of inward satisfaction our embryotomy case upon a top shelf, covered with the accumulated dust of years, as yet wholly unstained by the blood of the innocents; and we turn with a thrill of real pleasure to a well-worn Davis' forceps, which has proven itself a life-boat of safety to so many buds of humanity, and a harbinger of joyful deliverance to so many struggling and anxious mothers.

